# Kinetic analysis of dominant intraprostatic lesion of prostate cancer using quantitative dynamic [^18^F]DCFPyL-PET: comparison to [^18^F]fluorocholine-PET

**DOI:** 10.1186/s13550-020-00735-w

**Published:** 2021-01-04

**Authors:** Dae-Myoung Yang, Fiona Li, Glenn Bauman, Joseph Chin, Stephen Pautler, Madeleine Moussa, Irina Rachinsky, John Valliant, Ting-Yim Lee

**Affiliations:** 1 Department of Medical Biophysics, Schulich School of Medicine and Dentistry, University of Western Ontario, 1151 Richmond St, London, ON N6A 3K7 Canada; 2 Robarts Research Institute, University of Western Ontario, 1151 Richmond St, London, ON N6A 5B7 Canada; 3 Lawson Health Research Institute, 268 Grosvenor St, London, ON N6A 4V2 Canada; 4 Department of Oncology, Schulich School of Medicine and Dentistry, University of Western Ontario, 1151 Richmond St, London, ON N6A 3K7 Canada; 5 Pathology and Laboratory Medicine, London Health Sciences Centre, 800 Commissioners Rd E, London, ON N6A 5W9 Canada; 6 Centre for Probe Development and Commercialization, McMaster University, 1280 Main Street West, Hamilton, ON L8S 4K1 Canada

**Keywords:** Prostate cancer, [^18^F]fluorocholine, [^18^F]DCFPyL, Prostate-specific membrane antigen (PSMA), Dynamic positron emission tomography (PET), Kinetic analysis

## Abstract

**Purpose:**

Identification of the dominant intraprostatic lesion(s) (DILs) can facilitate diagnosis and treatment by targeting biologically significant intra-prostatic foci. A PSMA ligand, [^18^F]DCFPyL (2-(3-{1-carboxy-5-[(6-[^18^F]fluoro-pyridine-3-carbonyl)-amino]-pentyl}-ureido)-pentanedioic acid), is better than choline-based [^18^F]FCH (fluorocholine) in detecting and localizing DIL because of higher tumour contrast, particularly when imaging is delayed to 1 h post-injection. The goal of this study was to investigate whether the different imaging performance of [^18^F]FCH and [^18^F]DCFPyL can be explained by their kinetic behaviour in prostate cancer (PCa) and to evaluate whether DIL can be accurately detected and localized using a short duration dynamic positron emission tomography (PET).

**Methods:**

19 and 23 PCa patients were evaluated with dynamic [^18^F]DCFPyL and [^18^F]FCH PET, respectively. The dynamic imaging protocol with each tracer had a total imaging time of 22 min and consisted of multiple frames with acquisition times from 10 to 180 s. Tumour and benign tissue regions identified by sextant biopsy were compared using standardized uptake value (SUV) and tracer kinetic parameters from kinetic analysis of time-activity curves.

**Results:**

For [^18^F]DCFPyL, logistic regression identified *K*_i_ and *k*_4_ as the optimal model to discriminate tumour from benign tissue (84.2% sensitivity and 94.7% specificity), while only SUV was predictive for [^18^F]FCH (82.6% sensitivity and 87.0% specificity). The higher *k*_3_ (binding) of [^18^F]FCH than [^18^F]DCFPyL explains why [^18^F]FCH SUV can differentiate tumour from benign tissue within minutes of injection. Superior [^18^F]DCFPyL tumour contrast was due to the higher *k*_4_/*k*_3_ (more rapid washout) in benign tissue compared to tumour tissue.

**Conclusions:**

DIL was detected with good sensitivity and specificity using 22-min dynamic [^18^F]DCFPyL PET and avoids the need for delayed post-injection imaging timepoints. The dissimilar in vivo kinetic behaviour of [^18^F]DCFPyL and [^18^F]FCH could explain their different SUV images.

*Clinical Trial Registration* NCT04009174 (ClinicalTrials.gov).

## Introduction

Prostate cancer (PCa) is the most frequent cancer and one of the most common causes of cancer death in men in the USA and Canada [1, 2]. Positron emission tomography (PET) targeting prostate-specific membrane antigen (PSMA), a type II integral membrane protein, is generating significant interest recently. PSMA is a highly promising target for localizing and detecting PCa because it is overexpressed 100- to 1000-fold in malignant compared to benign prostate tissue [3]. Prior to PSMA, choline-based tracers that depend on increased phosphorylation of choline in lipid metabolism were widely used [4–6]. In comparison, radiotracers targeting PSMA, such as [^68^Ga]PSMA-11 and [^18^F]DCFPyL, afford better image quality and PCa detection rate than choline-based radiotracers [7–9]. Since previous clinical studies had shown that [^18^F]fluorocholine ([^18^F]FCH) PET cannot differentiate benign hyperplasia from PCa, it is not recommended for localizing PCa [10, 11]. However, PSMA PET requires a longer post-injection imaging time than [^18^F]FCH PET to achieve optimal standardized uptake value (SUV, a semi-quantitative measure of the lesion activity concentration normalized by the injected activity and body weight) contrast between PCa and background [12–17]. For example, it has been advocated that [^18^F]DCFPyL PET imaging should be performed at least 1 h post-injection compared with 7–30 min for [^18^F]FCH PET [8–11].

Even though PET SUV with a PSMA ligand, such as [^18^F]DCFPyL, showed high image quality and can visualize small prostate lesions with excellent sensitivity [18], it is a single time point measurement and is unable to completely describe the uptake of the tracer. The uptake of PET tracer in tissue, as measured by the SUV, is determined by the combined effects of three processes: tracer delivery via blood flow, exchange between vessels and tissue, and binding to and dissociation from the target. A single uptake measurement cannot differentiate among the 3 different processes [19]. Therefore, SUV is dependent on the time after injection when the measurement is made [20]. Moreover, these processes may vary among radiotracers, and they can be different among tumours of the same tumour type because of tumour heterogeneity. Instead, dynamic PET with its multiple time point measurements following injection is suited to dissect the processes involved in the distribution and uptake of radiotracers and it can provide additional metrics related to the target-specific molecular/metabolic processes for potential better differentiation of tumour from benign tissue.

Prior studies have made use of analysis methods that incorporate the above concepts of tracer transport (kinetic models) in modelling the time activity curves obtained from dynamic [^18^F]FCH and [^18^F]DCFPyL PET studies. [19, 21–23]. Schaefferkoetter et al. [21] found that with the tracer [^18^F]FCH, SUV and net uptake rate constant (*K*_i_) and influx rate constant (*K*_1_) using a reversible 2-tissue compartment (2T4k) model were found to be strong indicators of aggressive disease. Although SUV is simple to measure relative to kinetic analysis of dynamic PET, it cannot reliably quantify the transport (kinetics) of PCa-specific tracer as it is not correlated with either *K*_1_ of an irreversible binding one-tissue compartment model (1T1K) model [22] or *K*_i_ of a reversible binding two-tissue compartment model [23]. Moreover, for [^18^F]FCH, kinetic modelling results from a dynamic scan of 30 min reproduced those with a longer 60-min scan [21].

The goals of this study were twofold: first, to investigate whether the different behaviour of [^18^F]DCFPyL and [^18^F]FCH in PCa can be explained by kinetic analysis; second, to evaluate besides SUV which kinetic parameters derived from a 22-min dynamic [^18^F]FCH and [^18^F]DCFPyL PET study can localize and detect dominant intraprostatic lesion (DIL) identified by prostate sextant biopsy.

## Methods

### Patients

This prospective single-institution clinical study was approved by Institutional Research Ethics Board. All participants in this study provided written informed consent before the study. Enrolled subjects were men with untreated biopsy-proven localized PCa—3D-transrectal ultrasound (TRUS)-guided prostate sextant biopsy was done as standard of care within 3 months prior to the PET imaging to have sufficient interval time to prevent false positive. The inclusion criteria were as follows: aged 18 years or older; biopsy confirmed PCa; and suitable for and consenting to radical prostatectomy (RP) for treatment, or repeat biopsy as the standard of care. Only sextant biopsy data were used as tumour vs. benign reference, and no histopathology data from RP or repeat biopsies were used for this purpose. The exclusion criteria were as follows: had prior therapy including hormone therapy for PCa; use of 5-alpha reductase inhibitors—finasteride or dutasteride—within 6 months of study date; unable to comply with all pre-operative imaging; had sickle cell disease or other anaemias; impaired renal function (estimated GFR < 60 mL/min/1.73 m^2^); or residual bladder volume > 150 cc (determined by post-void ultrasound).

**Fig. 1:**
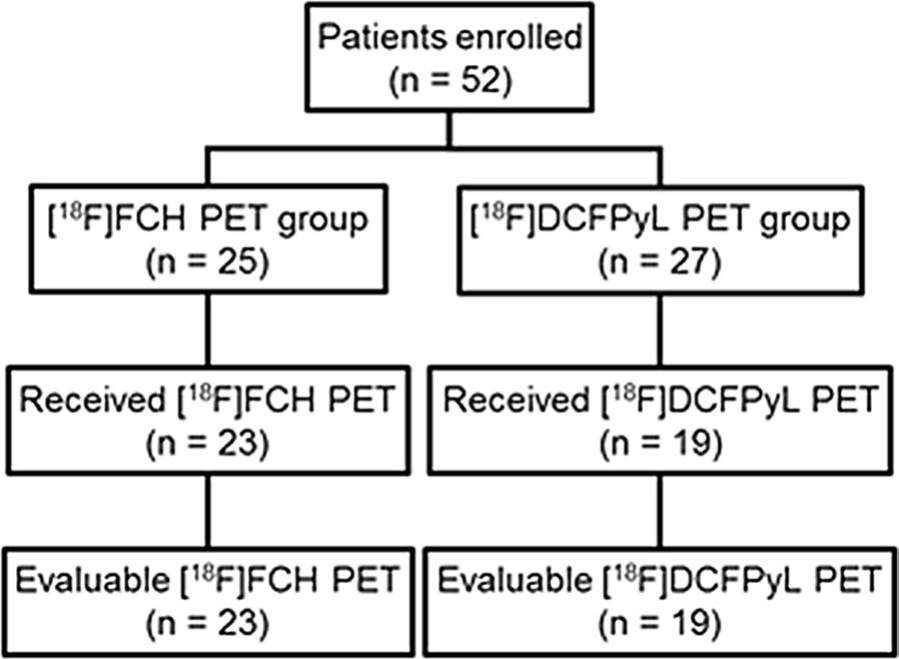
Study flow chart showing patient enrolment. FCH, fluorocholine; DCFPyL, 2-(3-{1-carboxy-5-[(6-[^18^F]fluoro-pyridine-3-carbonyl)-amino]-pentyl}-ureido)-pentanedioic acid

52 PCa patients were recruited into the study. The first 25 enrolled patients had dynamic [^18^F]FCH PET, while the last 27 patients had dynamic [^18^F]DCFPyL PET. However, 23 and 19 patients received [^18^F]FCH and [^18^F]DCFPyL PET, respectively, because of patients withdrawing from the study or failure of tracer production. Figure 1 shows the flow chart of the study.

### 3D-transrectal ultrasound (TRUS)-guided prostate sextant biopsy

All patients underwent systematic TRUS-guided biopsy (6- or 12-core) before the dynamic PET scan. The prostate sextants with the largest volume and/or highest grade cancer on biopsy were designated as harbouring a DIL. This information was extracted from the clinical biopsy report by one of the investigators (GB) and the involved sextants correlated with the SUV image from the dynamic PET scan (see next section).

### Dynamic PET imaging acquisition

Patients were instructed to fast, except for clear fluids, 12 h prior to the PET scan and to drink 500 mL of water 30 min prior to scan. No diuretics were used. Dynamic [^18^F]FCH and [^18^F]DCFPyL PET imaging was performed on a Discovery VCT (GE Healthcare, Waukesha, WI, USA) PET/CT scanner. A low-dose CT scan for attenuation correction and anatomical correlation was taken first with patients lying supine on the patient couch using the following technique: 50 mAs, 120kVp, without intravenous contrast, 47 slides with 3.27-mm spatial interval. The dynamic PET scan was set to cover the whole prostate and common iliac arteries. It was started simultaneously as the intravenous injection of 4 MBq/kg (median 381 MBq; range 280–538 MBq) [^18^F]FCH or 325 MBq (median 335 MBq; 280–348 MBq) [^18^F]DCFPyL without the patient moving from the CT scan position. The dynamic PET scan was acquired for 22 min under quiet breathing with variable frame lengths of 10 s (10 frames), 20 s (5), 40 s (4), 60 s (4) and 180 s (4). SUV of [^18^F]FCH or [^18^F]DCFPyL was measured by averaging the last four dynamic PET images, which was equivalent to 12 min of acquisition starting at 10 min post-injection. Both [^18^F]FCH and [^18^F]DCFPyL were obtained from the Centre for Probe Development and Commercialization (Hamilton, ON, Canada) at arm’s length.

### Dynamic PET imaging analysis

The dynamic PET images were reconstructed in activity concentration (kBq/mL) with use of the scanner sensitivity calibration factor that was routinely measured as quality control of the scanner. Since SUV is activity concentration normalized by injected dose and body weight, the reconstructed images when analyzed on an AW Workstation (AW4.7, GE Healthcare) can be displayed in units of activity concentration or SUV. In the DIL region indicated by pre-operative prostate sextant biopsy, regions of interest (ROIs) were drawn in all slices showing the largest lesion by thresholding SUV 50% of maximum SUV (SUV_max_). The alignment of the sextant biopsy report with the PET image was done manually and with consensus among the first author (DMY), radiation oncologist (GB) and nuclear medicine physician (IR). The same number of benign tissue ROIs as DIL ROIs was outlined in sextant locales not involved according to prostate sextant biopsy report. ROIs were confirmed by a radiation oncologist (GB) and a nuclear medicine physician (IR). For kinetic analysis, the arterial time-activity curve (TAC) was obtained from a region inside a common iliac artery. TACs for DIL and benign tissue were obtained by finding the area-weighted average of the mean activity in each DIL and benign ROI for all slices. The dynamic TACs of both tracers were analyzed using the flow-modified two-tissue compartment model (F2TCM), as described in a previous publication [19]. This model accounts for the combined effects of the three tracer uptake processes: tracer delivery via blood flow; bidirectional permeation of the blood-tissue barrier during tracer transit through vessels; and binding to and dissociation from the target. Kinetic parameters *K*_1_ (influx rate constant) in mL/min/g, *k*_2_ (efflux rate constant) in min^−1^, *k*_3_ (binding rate constant) in min^−1^, *k*_4_ (dissociation rate constant) in min^−1^, *K*_i_ = *K*_1_*k*_3_/(*k*_2_ + *k*_3_ + *k*_4_) (net uptake rate constant from plasma) in mL/min/g, DV = (*K*_1_/*k*_2_)(1 + *k*_3_/*k*_4_) (distribution volume) in mL/g and *k*_4_/*k*_3_ (normalized washout rate constant (inverse of binding potential)) were estimated by deconvolving the arterial TAC from the tissue TAC. A custom developed MATLAB program iteratively adjusted the model parameters until the sum of squared deviations of the fitted TAC, calculated as the convolution of the arterial TAC and the flow-scaled impulse residue function of the F2TCM, from the tissue TAC was minimized.

### Statistical analysis

All statistical analyses were performed using SPSS (IBM SPSS Statistics 23, IBM Analytics) for 2-sided testing with significance set at *P* < 0.05. For each PET tracer, the F2TCM parameters of DIL and benign prostatic tissue were compared using nonparametric Wilcoxon matched-pair signed-rank test. The kinetic parameters of [^18^F]FCH and [^18^F]DCFPyL were compared using the nonparametric Mann–Whitney *U* test. Logistic regression with backward selection was used to determine the most sensitive set of kinetic parameters to distinguish DIL from benign tissue for each tracer. From that analysis, sensitivity, specificity, positive predictive value (PPV) and negative predictive value (NPV) and receiver operating characteristic (ROC) curve of the dynamic [^18^F]FCH and [^18^F]DCFPyL PET imaging were assessed.

## Results

**Fig. 2:**
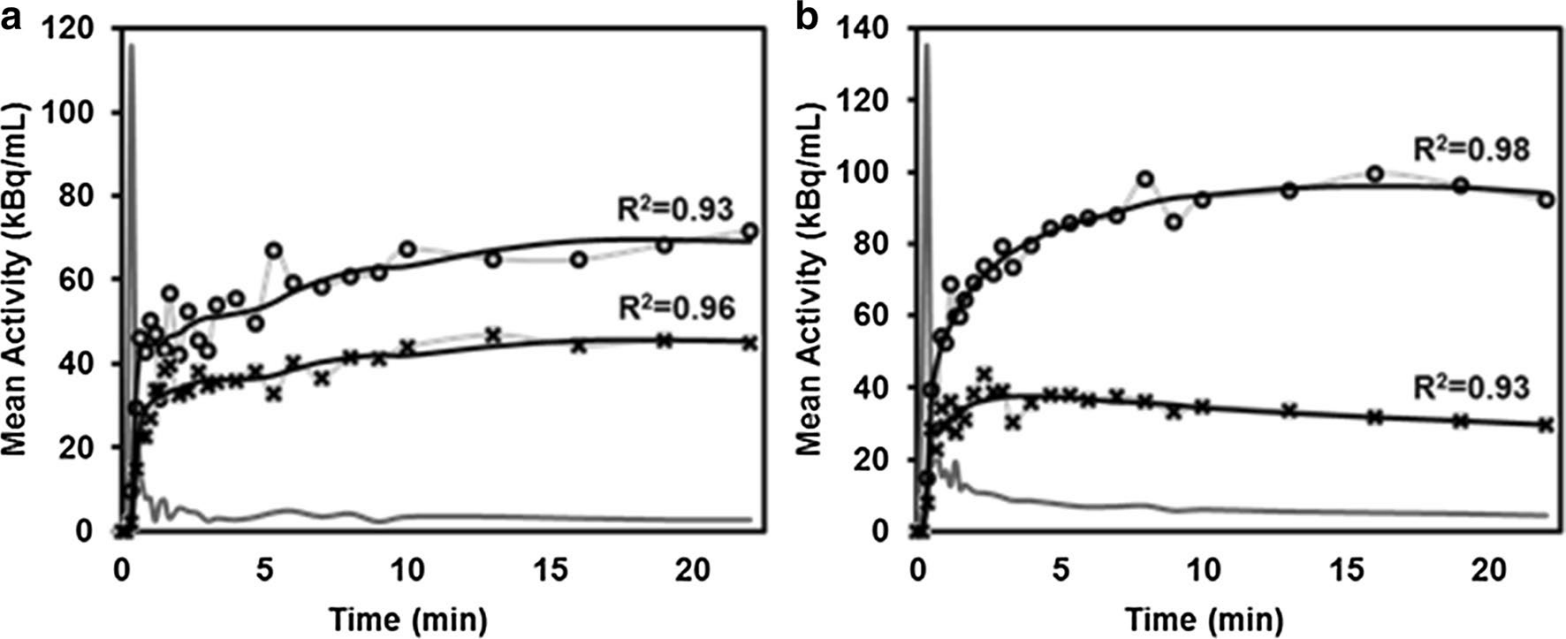
An iliac artery TAC (grey), the tumour TAC (light grey line with open circle marker, scaled up 4× to improve visibility) and the benign tissue TAC (light grey line with × marker, scaled up 4×) of a [^18^F]FCH patient (**a**) and a [^18^F]DCFPyL patient (**b**). Both patients had similar prostate cancer characteristics (PSA level 10.3 vs. 12.94 ng/mL; Gleason score 7 (3 + 4), the proportion of prostate involved with tumour 10%). The fitted curves (black) for both tracers using the F2TCM showed a strong correlation with measured TACs (*R*^2^ > 0.93). TAC, time-activity curve; FCH, fluorocholine; DCFPyL, 2-(3-{1-carboxy-5-[(6-[^18^F]fluoro-pyridine-3-carbonyl)-amino]-pentyl}-ureido)-pentanedioic acid; PSA, prostate-specific antigen; F2TCM, flow-modified standard two-extravascular-tissue compartment model

**Fig. 3:**
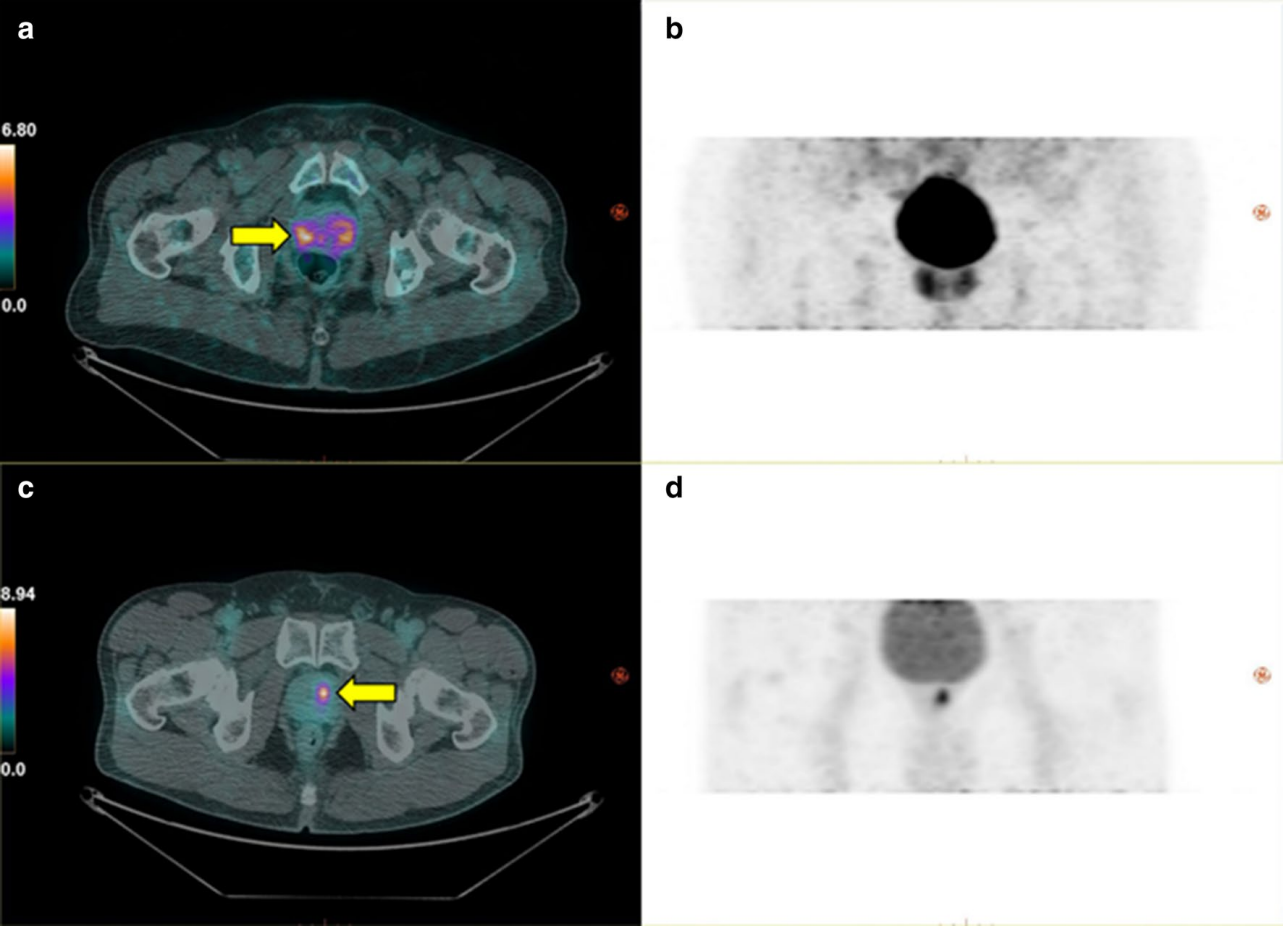
PET/CT images of the same patients as Fig. 2 showing maximum intensity projection of [^18^F]FCH PET SUV map in an axial view superimposed on CT (**a**) and in a coronal view by itself (**b**); the same two views for a [^18^F]DCFPyL study on another patient are shown in (**c**) and (**d**). The colour bar shows the scale of SUV. Localization of DIL (yellow arrow) with PET in both patients agreed with pre-operative prostate sextant biopsy. [^18^F]DCFPyL SUV map shows superior tumour contrast relative to benign prostatic tissue and better localization of DIL than [^18^F]FCH. PET, positron emission tomography; CT, computed tomography; FCH, fluorocholine; SUV, standardized uptake value; DCFPyL, 2-(3-{1-carboxy-5-[(6-[^18^F]fluoro-pyridine-3-carbonyl)-amino]-pentyl}-ureido)-pentanedioic acid; DIL, dominant intraprostatic lesion

23 patients (median age 62 years, range 49–76 years) and 19 patients (median age 63 years, range 53–69 years) received dynamic PET with [^18^F]FCH and [^18^F]DCFPyL, respectively (Fig. 1). The median pre-operative PSA level within 2 week before the day of the imaging session of [^18^F]FCH and [^18^F]DCFPyL cohort was 4.8 ng/mL (range 0.9–15.0 ng/mL) and 5.4 ng/mL (range 3.5–25.5 ng/mL), respectively. The characteristics of the two patient cohorts are listed in Table 1. Figure 2 shows the measured arterial and tissue TAC from a patient from each cohort, and the fit using the F2TCM. Figure 3 shows maximum intensity projections of PET images of the same patients, as in Fig. 2.

**Table 1:** Patient characteristics

	[^18^F]FCH (*n* = 23)	[^18^F]DCFPyL (*n* = 19)	*P* value
Age (y)	61.1 ± 6.9 (49–76)*	62.4 ± 4.7 (50–67)*	0.58
Weight (kg)	93.3 ± 14.3 (67–122)*	86.4 ± 17.1 (30–109)*	0.46
Height (cm)	175.7 ± 5.0 (168–185)*	177.1 ± 6.3 (165–191)*	0.37
PSA (ng/mL)	5.9 ± 3.4 (0.9–15.0)*	8.4 ± 5.8 (3.5–25.5)*	0.42
Histology—*n*(%)			1.00
Adenocarcinoma	23 (100)	19 (100)	
pT stage—*n*(%)			0.93
T2a	1 (4.3)	0 (0)	
T2c	12 (52.2)	11 (57.9)	
T3a	5 (21.7)	7 (36.8)	
T3b	5 (21.7)	1 (5.3)	
pN stage—*n*(%)			0.79
N0	19 (82.6)	19 (100)	
N1	1 (4.3)	0 (0)	
NX	3 (13.0)	0 (0)	
Gleason score—*n*(%)			0.94
6 (3 + 3)	2 (8.7)	1 (5.3)	
7 (3 + 4)	17 (69.6)	16 (84.2)	
7 (4 + 3)	4 (17.4)	2 (10.5)	
9 (5 + 4)	1 (4.3)	0 (0)	
Proportion of prostate involved by tumour (%)	14.3 ± 15.5 (1–80)*	13.7 ± 7.7 (5–30)*	0.49

Data are number of patients, with the percentage in parentheses unless otherwise indicated

FCH, fluorocholine; DCFPyL, 2-(3-{1-carboxy-5-[(6-[^18^F]fluoro-pyridine-3-carbonyl)-amino]-pentyl}-ureido)-pentanedioic acid; PSA, prostate-specific antigen

^*^Data are means ± standard deviation, with the range in parentheses

### Comparison of kinetic parameters between DIL and benign tissue parameters

**Fig. 4:**
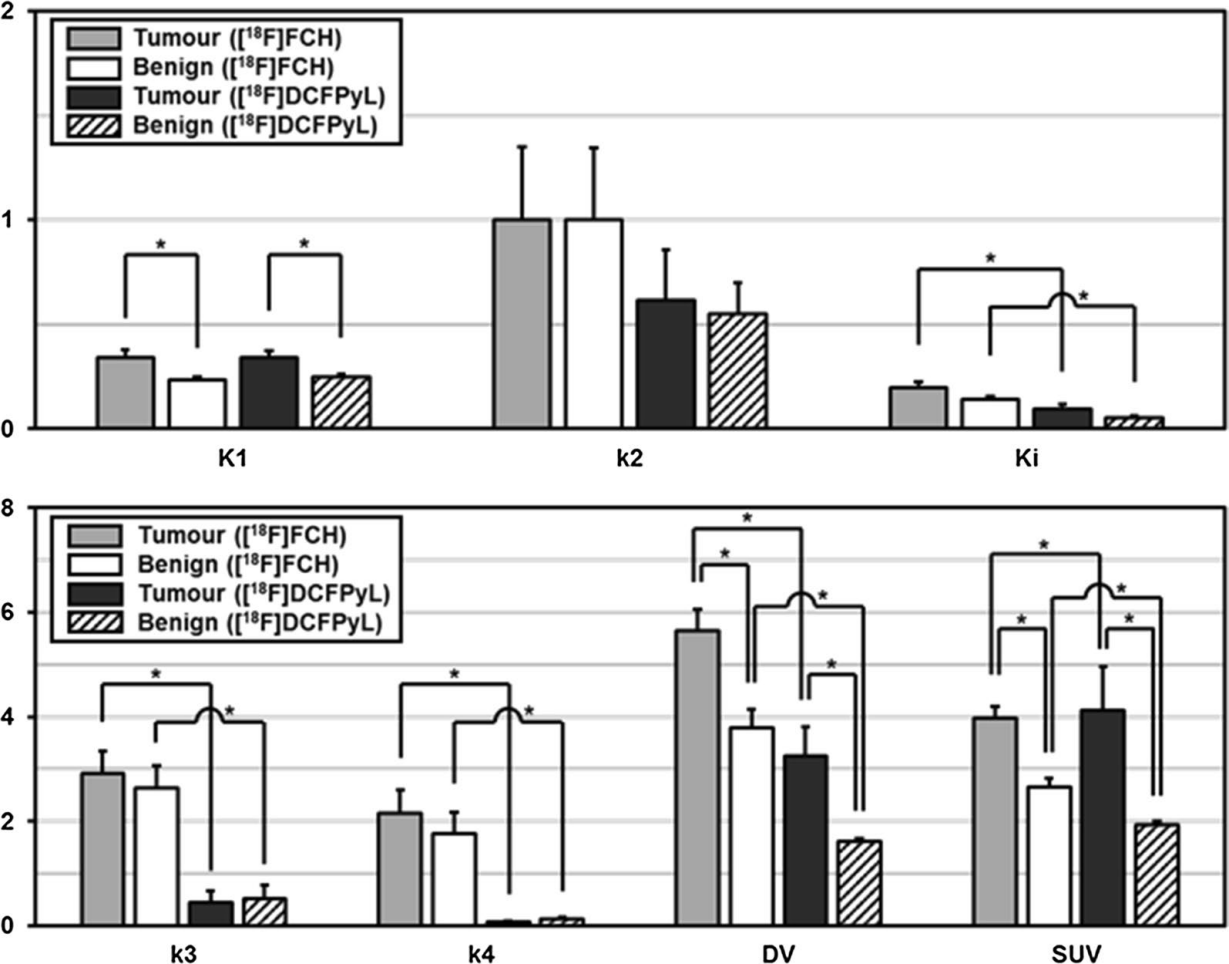
Comparison of [^18^F]FCH and [^18^F]DCFPyL F2TCM model parameters in DIL and benign tissue. Significant difference (*P* < 0.05) is marked with *. FCH, fluorocholine; DCFPyL, 2-(3-{1-carboxy-5-[(6-[^18^F]fluoro-pyridine-3-carbonyl)-amino]-pentyl}-ureido)-pentanedioic acid; F2TCM, flow-modified standard two-extravascular-tissue compartment model; DIL, dominant intraprostatic lesion; *K*_1_, influx rate; *k*_2_, efflux rate constant; *k*_3_, binding rate constant; *k*_4_, dissociation rate constant; *K*_i_, net uptake rate constant from plasma; DV, distribution volume; SUV, standardized uptake value

Kinetic parameters of DIL were compared with those of benign tissue in Fig. 4. For [^18^F]FCH cohort, significant differences in median value of *K*_1_ (0.27 vs. 0.23 mL/min/g; *P* < 0.001), SUV (3.88 vs. 2.75 g/mL; *P* < 0.001) and DV (6.07 vs. 4.31 mL/g; *P* = 0.04) were found. The median values of the same three parameters and also *k*_4_/*k*_3_ were different between DIL and benign tissue for [^18^F]DCFPyL cohort—*K*_1_ (0.30 vs. 0.24 mL/min/g; *P* = 0.02), SUV (2.76 vs. 1.96 g/mL; *P* < 0.001), DV (3.89 vs. 1.42 mL/g; *P* = 0.01) and *k*_4_/*k*_3_ (0.41 vs. 0.69 unitless; *P* = 0.03).

### Diagnostic performance for detecting and localizing DIL using PET kinetic parameters

**Fig. 5:**
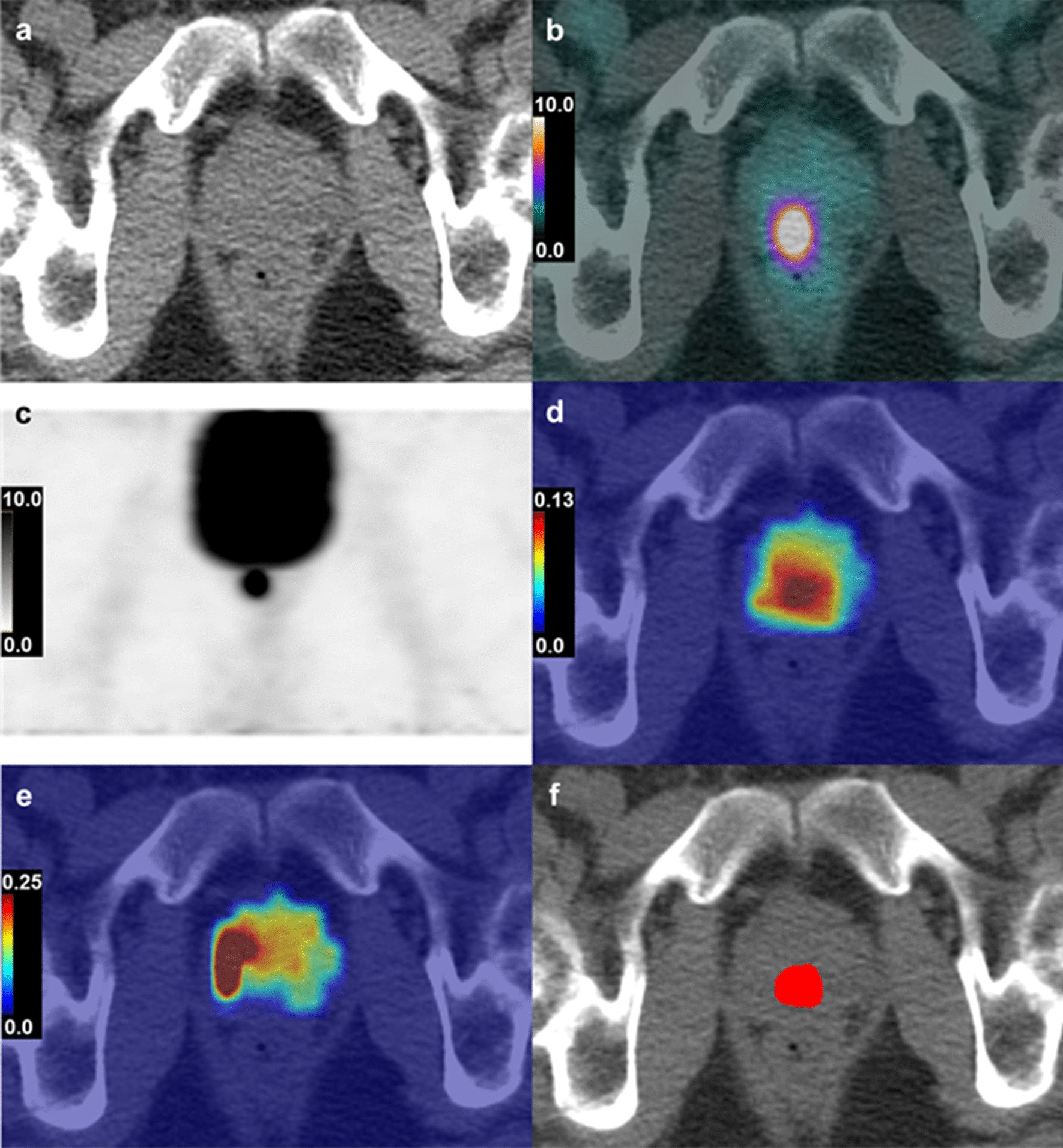
Axial CT image (**a**), axial [^18^F]DCFPyL SUV map (range 0–10.0 g/mL) superimposed on CT (**b**), coronal [^18^F]DCFPyL PET (range 0–10.0 g/mL) (**c**), K_i_ parametric map (**d**), *k*_4_ parametric map (**e**) and localized tumour in red using the classifier from logistic regression (**f**), in a 64-year-old man with PCa (Gleason score 7 (3 + 4)). Pre-op PSA was 8.17 ng/mL. Mean SUV of the tumour ROI was 13.4 g/mL. CT, computed tomography; DCFPyL, 2-(3-{1-carboxy-5-[(6-[^18^F]fluoro-pyridine-3-carbonyl)-amino]-pentyl}-ureido)-pentanedioic acid; SUV, standardized uptake value; PET, positron emission tomography; *K*_i_, net uptake rate constant from plasma; *k*_4_, dissociation rate constant; PCa, prostate cancer; PSA, prostate-specific antigen; ROI, region of interest

For the [^18^F]FCH group (*n* = 23), using logistic regression with backward elimination of all parameters having a univariable logical regression *P* < 0.2, the most sensitive model of PET parameters for identifying DIL from benign tissue consisted of SUV alone (*P* < 0.001)—sensitivity 82.6%, specificity 87.0%, PPV 86.4%, NPV 83.3%, and area under the ROC curve (AUC) 0.88. For [^18^F]DCFPyL, logistic regression with backward elimination of all parameters having a univariable logical regression *P* < 0.2 yielded the combination of *K*_i_ and *k*_4_ as the most sensitive model (*P* < 0.001) that distinguished tumour to benign tissue—sensitivity 84.2%, specificity 94.7%, PPV 94.1%, NPV 85.7%, and AUC 0.93. A representative case of [^18^F]DCFPyL SUV, *K*_i_ and *k*_4_ parametric maps is shown in Fig. 5.

### ***Kinetic parameters of [***^***18***^***F]FCH vs [***^***18***^***F]DCFPyL***

**Fig. 6:**
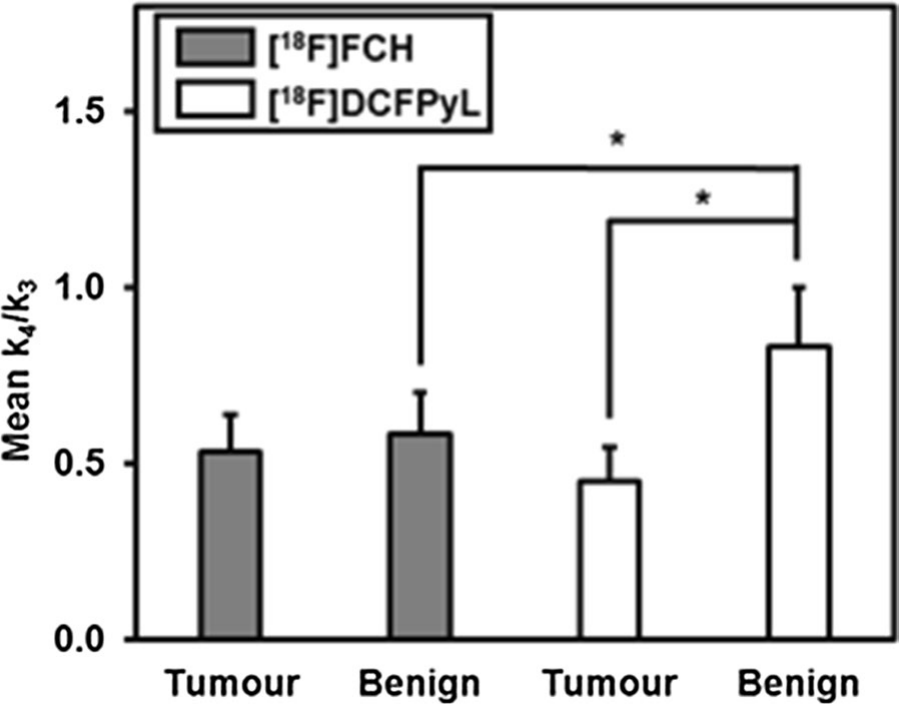
Comparison of normalized washout rate constant (*k*_4_/*k*_3_) (mean ± SEM) of [^18^F]FCH and [^18^F]DCFPyL in both tumour and benign tissues, significant differences are marked with (*), *P* < 0.05. *k*_4_/*k*_3_, Normalized washout rate constant (inverse of binding potential); SEM, standard error of the mean; FCH, fluorocholine; DCFPyL, 2-(3-{1-carboxy-5-[(6-[^18^F]fluoro-pyridine-3-carbonyl)-amino]-pentyl}-ureido)-pentanedioic acid

The in vivo behaviour of [^18^F]FCH in 23 patients and [^18^F]DCFPyL in 19 patients were compared using kinetic parameters estimated from dynamic PET (see Fig. 4). There were no significant differences in *K*_1_ and *k*_2_ between the two tracers in both tumour and benign tissue. For [^18^F]FCH versus [^18^F]DCFPyL (mean ± SD) in tumour tissue, there was a significant difference in SUV (3.98 ± 1.08 vs. 4.12 ± 3.64; *P* = 0.01), *k*_3_ (2.91 ± 2.03 vs. 0.44 ± 1.04 min^−1^; *P* = 0.001), *k*_4_ (2.16 ± 2.13 vs. 0.08 ± 0.07 min^−1^; *P* < 0.001), *K*_i_ (0.20 ± 0.14 vs. 0.10 ± 0.10 mL/min/g; *P* = 0.002) and DV (4.50 ± 3.83 vs. 3.40 ± 2.26 mL/g; *P* = 0.03). In benign tissue, there was significant difference in SUV (2.66 ± 0.81 vs. 1.92 ± 0.37 g/mL; *P* = 0.001), *k*_3_ (2.63 ± 2.06 vs. 0.51 ± 1.16 min^−1^; *P* < 0.001), *k*_4_ (1.75 ± 2.03 vs. 0.13 ± 0.12 min^−1^; *P* = 0.004), *K*_i_ (0.14 ± 0.07 vs. 0.05 ± 0.04 mL/min/g; *P* < 0.001) and DV (3.01 ± 2.12 vs. 1.50 ± 0.55 mL/g; *P* = 0.001). Figure 6 shows the normalized washout rate constant (*k*_4_/*k*_3_) of [^18^F]FCH and [^18^F]DCFPyL in DIL and benign tissue. This washout constant or inverse of binding potential was 1.86-fold higher in benign tissue than in tumour for [^18^F]DCFPyL (*P* < 0.05); however, for [^18^F]FCH, it was similar for both tissue types. In addition, for benign tissue, the normalized washout constant was higher for [^18^F]DCFPyL.

## Discussion

In this study, we estimated the F2TCM parameters of [^18^F]FCH and [^18^F]DCFPyL from dynamic PET studies of PCa patients and investigated whether these kinetic parameters can differentiate DIL from benign tissue and explain the different SUV image characteristics observed with the two tracers.

Logistic regression with backward elimination of variables showed that the combination of *K*_i_ and *k*_4_ and SUV alone were sensitive models for localizing and differentiating tumour from benign prostatic tissue with [^18^F]DCFPyL and [^18^F]FCH, respectively. The former tracer was more accurate according to AUC (0.93 vs. 0.88; *P* < 0.001 for both). Previous experience with [^18^F]FCH illustrated that it is challenging to localize the DIL without the prostate biopsy report because the tracer is not highly specific to PCa [10, 11, 24, 25]. Two prior studies on PCa-specific tracers, like ours, showed that SUV cannot substitute for kinetic analysis which has the advantage of taking into account of the plasma input function as well as the blood volume contribution, both of which are folded into the SUV calculation [22, 23]. However, in selected patients who have undergone multiple TRUS-guided biopsies with negative findings, [^18^F]FCH SUV map could be helpful and contribute valuable additional information for detection of the primary tumour [11].

For [^18^F]DCFPyL, *K*_i_ and *k*_4_ were more sensitive than the semi-quantitative SUV used routinely in the clinic. Previous studies had demonstrated that optimal image qualities (SUV tumour to background ratio) were achievable at 60–120 min post-injection [16, 17] due to reduction in nonspecific background [9, 26]. However, in this study, a 22-min dynamic PET acquisition with kinetic analysis to derive *K*_i_ and *k*_4_ of the F2TCM was able to differentiate tumour from benign tissue. Therefore, use of dynamic [^18^F]DCFPyL imaging may improve the efficiency of DIL imaging with PET by eliminating the 1–2 h wait time between injection and SUV imaging; moreover, different study also suggested that *K*_i_ with fixed *k*_4_ was preferred reference parameter in metastasized PCa with [^18^F]DCFPyL [23].

The observed better contrast between DIL and benign tissue with [^18^F]DCFPyL than [^18^F]FCH and that this contrast has been reported to increase with time [16, 17] could be explained by the different kinetic behaviour of the two tracers. Median *k*_3_, *k*_4_, *K*_i_ and SUV values of [^18^F]DCFPyL were smaller than those of [^18^F]FCH for both tumour and benign tissue. These differences in kinetics could explain why with [^18^F]FCH imaging can start soon after the tracer injection because [^18^F]FCH bound and dissociated more quickly than [^18^F]DCFPyL [10, 12–14, 27]. Higher normalized washout rate constant (*k*_4_/*k*_3_) indicates rapid washout relative to binding in the tissue. Figure 6 shows that for [^18^F]DCFPyL, normalized washout rate constant was significantly higher in benign tissue than in tumour (*P* < 0.05), while for [^18^F]FCH, this rate constant was not significantly different. Therefore, the contrast between tumour (DIL) and benign tissue would increase over time with [^18^F]DCFPyL, while the same contrast would not change over time with [^18^F]FCH. Taken together, the kinetic analysis suggests that with [^18^F]FCH, SUV imaging can be done soon after injection but contrast between tumour and benign tissue does not improved over time. In contrast, with [^18^F]DCFPyL, to optimize contrast between tumour and benign tissue, SUV imaging has to be delayed, as literature suggested, to 1–2 h post-injection. However, with kinetic analysis of dynamic [^18^F]DCFPyL PET acquired over 22 min from injection, the combination of *K*_i_ and *k*_4_ from the analysis could identify DIL with high accuracy (AUC = 0.93) avoiding the need for delayed imaging.

There are limitations with our study. First, the study investigated only a limited number of patients. The results warrant future external validation with larger number of patients. Second, dynamic [^18^F]DCFPyL imaging was limited to 22 min which precluded comparison with SUV at 1–2 h post-injection for separating tumour (DIL) from benign tissue. Third, burden of disease on sextant biopsy was used for defining DIL locations and this may be subject to sampling error. Fourth, ideally the performance of [^18^F]FCH and [^18^F]DCFPyL would be compared in the same patient group but availability of the tracer (and concerns about cumulative radiation dose) precluded such a comparison. The two patient cohorts were accrued sequentially on the same research protocol (same eligibility), and both cohorts had similar clinical characteristics (Table 1) reducing possible bias [26, 28].

## Conclusions

Patients with PCa were studied with dynamic [^18^F]FCH PET and dynamic [^18^F]DCFPyL PET over a short acquisition time of 22 min. Multiple kinetic parameters were derived with the custom developed F2TCM from the dynamic studies and compared for distinguishing tumour from benign tissue. Among all the [^18^F]FCH PET and [^18^F]DCFPyL parameters investigated, the logistic regression model based on *K*_i_ (net uptake rate constant from plasma) and *k*_4_ (dissociation rate constant from binding) of [^18^F]DCFPyL was the most accurate in identifying DIL containing sextants on prostate biopsy and these findings support the incorporation of dynamic imaging sequences into PET/CT protocols using [^18^F]DCFPyL.

## Data Availability

Clinical details of the cases and laboratory and imaging data restricted to non-identifying data owing to privacy concerns, can be requested by e-mail from the corresponding author, who will handle all requests.
